# Author’s response to the letter “Japanese clinical practice guidelines for rehabilitation in critically ill patients 2023 (J-ReCIP 2023)”

**DOI:** 10.1186/s40560-024-00751-1

**Published:** 2024-09-26

**Authors:** Fumihito Kasai, Yuki Iida, Takeshi Unoki

**Affiliations:** 1 Department of Rehabilitation Medicine, Showa University School of Medicine, Tokyo, Japan; 2 Department of Physical Therapy, Faculty of Health and Medical Sciences, Aichi Shukutoku University, Nagakute, Japan; 3 Department of Acute and Critical Care Nursing, School of Nursing, Sapporo City University, Sapporo, Japan

**Keywords:** Rehabilitation, Guidelines, Intensive care, Dysphagia

## Abstract

Recently, a Letter to the Editor critiquing the recommendations of the Japanese Clinical Practice Guidelines for Rehabilitation in Critically Ill Patients, 2023, was published. The comment centered on the recommendation, "Weak recommendation against the use of endoscopy-based management (GRADE 2D: certainty of evidence = 'very low')" for the clinical question, "Should critically ill patients be managed based on video endoscopic assessment of swallowing?" In response, we outline the rationale behind our recommendations and their clinical implications.

Thank you for submitting your letter [1] concerning the Japanese Clinical Practice Guidelines for the Rehabilitation of Critically Ill Patients 2023 (J-ReCIP 2023) that we published [2]. Your letter is thought-provoking and provides a precise list of important issues. Your enthusiasm for providing swallowing care in the ICU is commendable, and we believe that your logical aspects will accelerate the development of this field of research.

We agree that Flexible Endoscopic Evaluation of Swallowing (FEES), the gold standard for evaluating swallowing function, exhibits high diagnostic accuracy in critically ill patients in the ICU. Assessment of post-extubation swallowing using FEES using this protocol has shown a sensitivity and specificity of 97% and 49%, respectively [3]. However, our goal is to create guidelines based on a high level of evidence, which leads us to adopt only randomized controlled trials (RCTs) as the basis for individual research papers. In the absence of RCTs, the committee has debated whether to include non-randomized studies or qualitative studies, but ultimately decided to include only RCTs. This decision aligns with the conclusions of Barquist et al. [4], who stated that an FEES examination for all patients with > 48 h of endotracheal intubation did not reduce the rate of post-aspiration pneumonia. Thus, we cannot recommend such examinations for all patients in environments where they are carefully assessed and monitored.” In the ICU, aspiration pneumonia significantly affects prognosis; therefore, testing methods proven to be safe and effective should be used. One important point is that these guidelines provide recommendations on the impact of video-endoscopy-based management and not on the accuracy of swallowing function assessment. We used mortality, occurrence of pneumonia, feeding status, and time from extubation to oral intake as outcomes in this systematic review of clinical questions. We have set mortality as the most important outcome (9 points) and the occurrence of pneumonia as the second most important outcome (8 points). Our guideline-development committee adhered to the GRADE approach (Grading of Recommendations, Assessment, Development, and Evaluation), which employs a rigorous methodology to develop the following recommendation for managing critically ill patients in the ICU: a weak recommendation against the use of endoscopy-based management for all patients (GRADE 2D: certainty of evidence = "very low").

Based on the current evidence, our guidelines weakly recommends non-routine video-endoscopy-based management for all patients admitted to the ICU. However, this does not preclude VE-based management, which considers individual patient backgrounds. It is important to note that the recommended results may have been different if study designs other than RCTs were included. It is not our intention that FEES, with its high accuracy in detecting aspiration risk, should be discontinued in ICUs. Notably, if FEES-based management is to be considered, it should be performed by skilled medical personnel in adequate facilities, with careful consideration of the occurrence of pneumonia and adverse events. We hope that these guidelines will serve as an impetus for conducting RCTs and accumulating evidence to determine the effectiveness of FEES-based management of critically ill patients.

## Data Availability

Not applicable.

## Abbreviations

J-ReCIP 2023: The Japanese Clinical Practice Guidelines for Rehabilitation in Critically Ill Patients
FEES: Flexible Endoscopic Evaluation of Swallowing (video-endoscopic examination of swallowing in our guidelines)
GRADE: Grading of Recommendations Assessment, Development, and Evaluation
